# Continuous three-dimensional transesophageal echocardiography and deep learning for perioperative monitoring of left ventricular longitudinal function

**DOI:** 10.1093/ehjimp/qyaf052

**Published:** 2025-05-02

**Authors:** Jinyang Yu, Anders Austlid Taskén, Erik Andreas Rye Berg, Tomas Dybos Tannvik, Katrine Hordnes Slagsvold, Idar Kirkeby–Garstad, Bjørnar Grenne, Gabriel Kiss, Svend Aakhus

**Affiliations:** Department of Circulation and Medical Imaging, Fakultet for medisin og helsevitenskap, Norwegian University of Science and Technology, Postboks 8905, 7491 Trondheim, Norway; Clinic of Cardiology St. Olav’s Hospital, Trondheim University Hospital, Prinsesse Kristinas gate 3, 7030 Trondheim, Norway; Department of Anesthesia and Intensive Care, St. Olav’s Hospital, Trondheim University Hospital, Prinsesse Kristinas gate 3, 7030 Trondheim, Norway; Department of Computer Science, Norwegian University of Science and Technology, Trondheim, Norway; Department of Circulation and Medical Imaging, Fakultet for medisin og helsevitenskap, Norwegian University of Science and Technology, Postboks 8905, 7491 Trondheim, Norway; Clinic of Cardiology St. Olav’s Hospital, Trondheim University Hospital, Prinsesse Kristinas gate 3, 7030 Trondheim, Norway; Department of Anesthesia and Intensive Care, St. Olav’s Hospital, Trondheim University Hospital, Prinsesse Kristinas gate 3, 7030 Trondheim, Norway; Department of Circulation and Medical Imaging, Fakultet for medisin og helsevitenskap, Norwegian University of Science and Technology, Postboks 8905, 7491 Trondheim, Norway; Clinic of Cardiothoracic Surgery, St. Olav’s Hospital, Trondheim University Hospital, Trondheim, Norway; Department of Circulation and Medical Imaging, Fakultet for medisin og helsevitenskap, Norwegian University of Science and Technology, Postboks 8905, 7491 Trondheim, Norway; Department of Circulation and Medical Imaging, Fakultet for medisin og helsevitenskap, Norwegian University of Science and Technology, Postboks 8905, 7491 Trondheim, Norway; Clinic of Cardiology St. Olav’s Hospital, Trondheim University Hospital, Prinsesse Kristinas gate 3, 7030 Trondheim, Norway; Centre of Innovative Ultrasound Solutions, Faculty of Medicine and Health Science, Norwegian University of Science and Technology, Trondheim, Norway; Department of Computer Science, Norwegian University of Science and Technology, Trondheim, Norway; Department of Circulation and Medical Imaging, Fakultet for medisin og helsevitenskap, Norwegian University of Science and Technology, Postboks 8905, 7491 Trondheim, Norway; Clinic of Cardiology St. Olav’s Hospital, Trondheim University Hospital, Prinsesse Kristinas gate 3, 7030 Trondheim, Norway

**Keywords:** autoMAPSE, deep learning, 3D transesophageal echocardiography, MAPSE, hemodynamic monitoring, LV function

## Abstract

**Aims:**

Continuous monitoring of left ventricular (LV) function may improve cardiopulmonary management. Therefore, we have developed *3D autoMAPSE*, a novel method that combines 3D transesophageal echocardiography and deep learning to automatically measure mitral annular plane systolic excursion (MAPSE). We hypothesized that 3D autoMAPSE could provide continuous monitoring of LV function in perioperative patients.

**Methods and results:**

This prospective observational study included 50 adult intensive care patients after cardiac surgery. Single-beat full-volume 3D recordings were obtained every 5 min over a 120-min period using a hands-free method that simulated continuous monitoring with transesophageal echocardiography. Precision and agreement with manual MAPSE were determined by a test-retest study design during hemodynamic stability. Our results show that continuous monitoring by 3D autoMAPSE had excellent feasibility (99%). It analysed 10 cycles instantaneously (55 ± 15 s) with high precision (least significant change 1.6 mm). 3D autoMAPSE had adequate agreement with manual MAPSE (bias –1.4 mm, limits of agreement −4.0 to 1.2 mm). Continuous 3D autoMAPSE was associated with both N-terminal pro B-type natriuretic peptide (*rho* = −0.37, *P* = 0.008) and high-sensitivity troponin-T (*rho* = −0.28, *P* = 0.047). This association was slightly stronger than for LV ejection fraction or any other single echocardiographic measurement.

**Conclusion:**

3D autoMAPSE provided continuous monitoring of LV function in perioperative patients by obtaining highly feasible and precise measurements that agree with manual echocardiography and postoperative biomarkers. Thus, continuous 3D autoMAPSE may be an attractive complement to hemodynamic monitoring that can aid cardiopulmonary management.

## Graphical Abstract

**Graphical Abstract:**
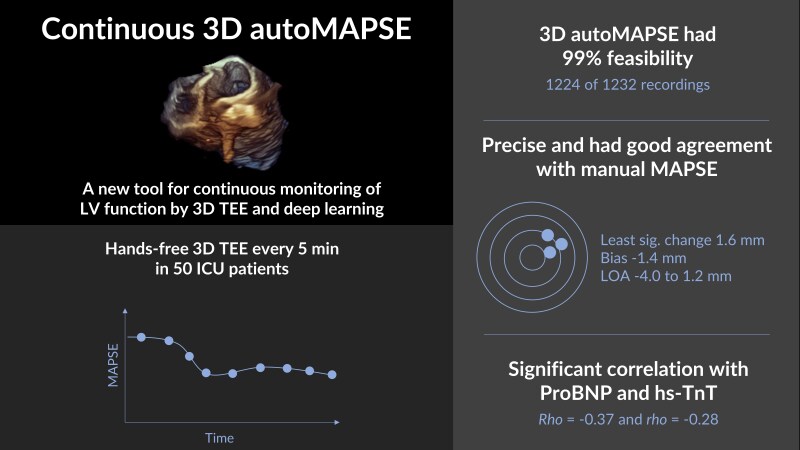

## Introduction

Left ventricular (LV) function, a vital determinant for cardiopulmonary management and prognosis,^1,2^ can deteriorate unpredictably in perioperative patients.^3^ Changes in related hemodynamic variables, mainly arterial pressure and cardiac output, are effectively detected by continuous hemodynamic monitoring. Unfortunately, these variables fail to detect LV dysfunction before the development of shock.^4^ Detection of LV dysfunction requires echocardiography,^4^ but this time-consuming modality is too slow and laborious for continuous monitoring. Likewise, although postoperative high-sensitivity troponin-T (hs-TnT)^5^ and N-terminal pro B-type natriuretic peptide (ProBNP)^6^ are prognostically important after cardiac surgery, they mainly reflect well-established LV dysfunction or injury. As a result, the detection of LV dysfunction is often delayed, which may allow the establishment of potentially permanent complications.

To monitor LV function continuously, we have combined transesophageal echocardiography (TEE) with deep learning for automatic measurement of mitral annular plane systolic excursion (MAPSE) in three-dimensional (3D) images, i.e. 3D autoMAPSE.^7^ This method has three advantages. First, MAPSE quantifies LV longitudinal function^8^ and is, like global longitudinal strain,^9^ more sensitive than LV ejection fraction (LVEF) in detecting LV dysfunction.^8,10^ Second, the mitral annulus is distinct from surrounding structures and lies close to the TEE probe, making deep learning detection highly feasible.^11–14^ Third, the esophagus stabilizes the TEE probe, which allows frequent hands-free image acquisition.^14^ The initial study on 3D autoMAPSE has shown promise,^7^ but the adequacy of the image quality and temporal resolution for continuous monitoring remains to be tested.

We hypothesized that 3D autoMAPSE could provide continuous monitoring of LV function in perioperative patients and thereby also reflect postoperative cardiac biomarkers. Thus, this prospective study aimed to determine the feasibility and precision of 3D autoMAPSE and to validate the method against manual echocardiographic measurements and postoperative cardiac biomarkers.

## Methods

### Patients and ethics

This prospective single-centre study included 50 adult patients above 18 years of age scheduled for any on-pump cardiac surgery with intraoperative TEE. All patients provided written informed consent before participating. They were included between October 2022 and March 2023 at St. Olav’s University Hospital, Trondheim, Norway. Exclusion criteria were non-consent and clinical contraindications to TEE.^15^ Due to study resources, we included a maximum of one patient per day, and patients having surgery off-hours were not included. The study was designed not to interfere with routine clinical care. We have previously reported other results from this material in studies for autoMAPSE using two-dimensional images (2D autoMAPSE).^11,14^

The study conforms with the principles of the Declaration of Helsinki and was approved by the Regional Committee for Ethics in Medicine. We adhered to the STROBE checklist^16^ when composing this manuscript.

### Hands-free echocardiographic image acquisition

**Figure 1:**
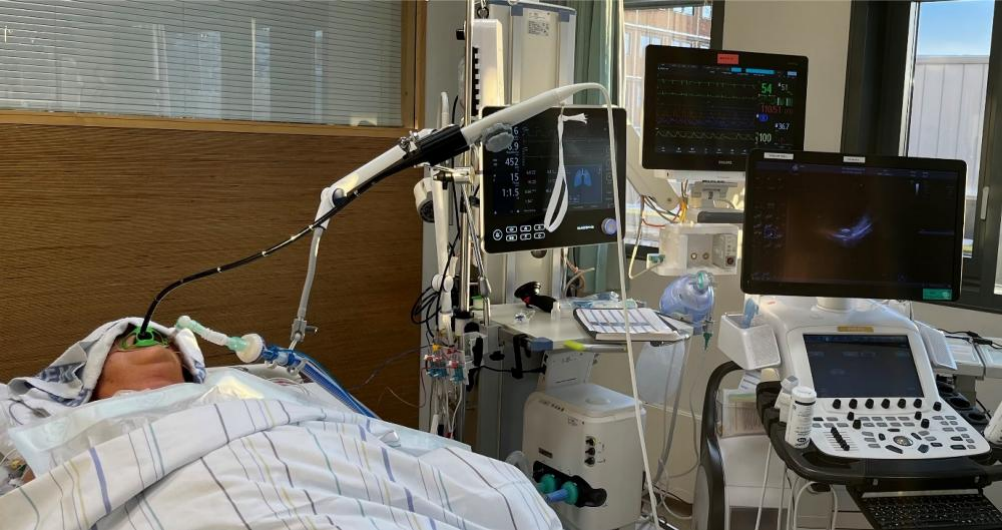
Continuous monitoring using hands-free transesophageal echocardiography. This photograph shows the transesophageal probe in a probe holder for hands-free image acquisition. The patient provided consent before being photographed.

All hands-free images were acquired in intubated patients upon arrival at the intensive care unit (ICU). For these images, single-beat full-volume 3D recordings were obtained using an E95 scanner and a 6VT probe (GE Healthcare, Horten, Norway). A researcher with training in 3D TEE and 3 years of clinical anaesthesiology (J.Y.) obtained all the images. The images were aimed at the mitral annulus, the depth was increased to include the LV apex, the volume size was maximized to 90 times 90 degrees, and the temporal resolution was adjusted to at least 15 volumes per second. Each recording comprised 10 cardiac cycles in order to improve the precision of the measurements. A probe holder was used for hands-free image acquisition (*Figure 1*). The probe tip was maintained in a passive position to minimize the risk of esophageal trauma.

**Figure 2:**
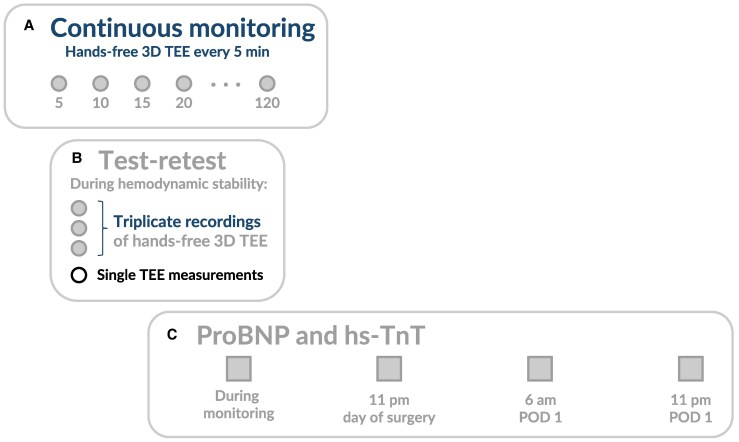
Overview of the study design and time points of measurements. (*A*) Continuous monitoring using 3D autoMAPSE. Each grey circle indicates a hands-free 3D TEE recording obtained every 5 min. (*B*) Test-retest design and single TEE measurements during hemodynamic stability. The grey circles indicate rapidly obtained triplicate recordings of hands-free 3D TEE images; 3D manual MAPSE was also measured in these images. The open black circle indicates the manual acquisition and analysis of conventional TEE images. (*C*) The grey squares indicate the four time points where ProBNP and hs-TnT were measured. 3D autoMAPSE, automatic measurements of mitral annular plane systolic excursion by 3D transesophageal echocardiography; hs-TnT, high-sensitivity troponin-T; MAPSE, mitral annular plane systolic excursion; POD, postoperative Day; ProBNP, N-terminal pro B-type natriuretic peptide; TEE, transesophageal echocardiography.

This hands-free technique simulated semi-continuous monitoring by manually recording 3D images every 5 min for 120 min (*Figure 2A*). Continuous 3D imaging produces heat quickly, so we limited the image acquisitions to every 5 min to minimize the risk of thermal injury. Manual adjustments of the probe were scheduled every 20 min. Unscheduled adjustments were made if the left ventricle was dislocated from the image.

In a test-retest protocol, we recorded triplicate recordings in rapid succession during a period of stable mean arterial pressure, heart rate, and drug infusion (*Figure 2B*); each recording comprised 10 cardiac cycles. These triplicate recordings were used to assess the precision of 3D autoMAPSE and for comparison with manual measurements of MAPSE.

### 3D autoMAPSE measurements

**Figure 3:**
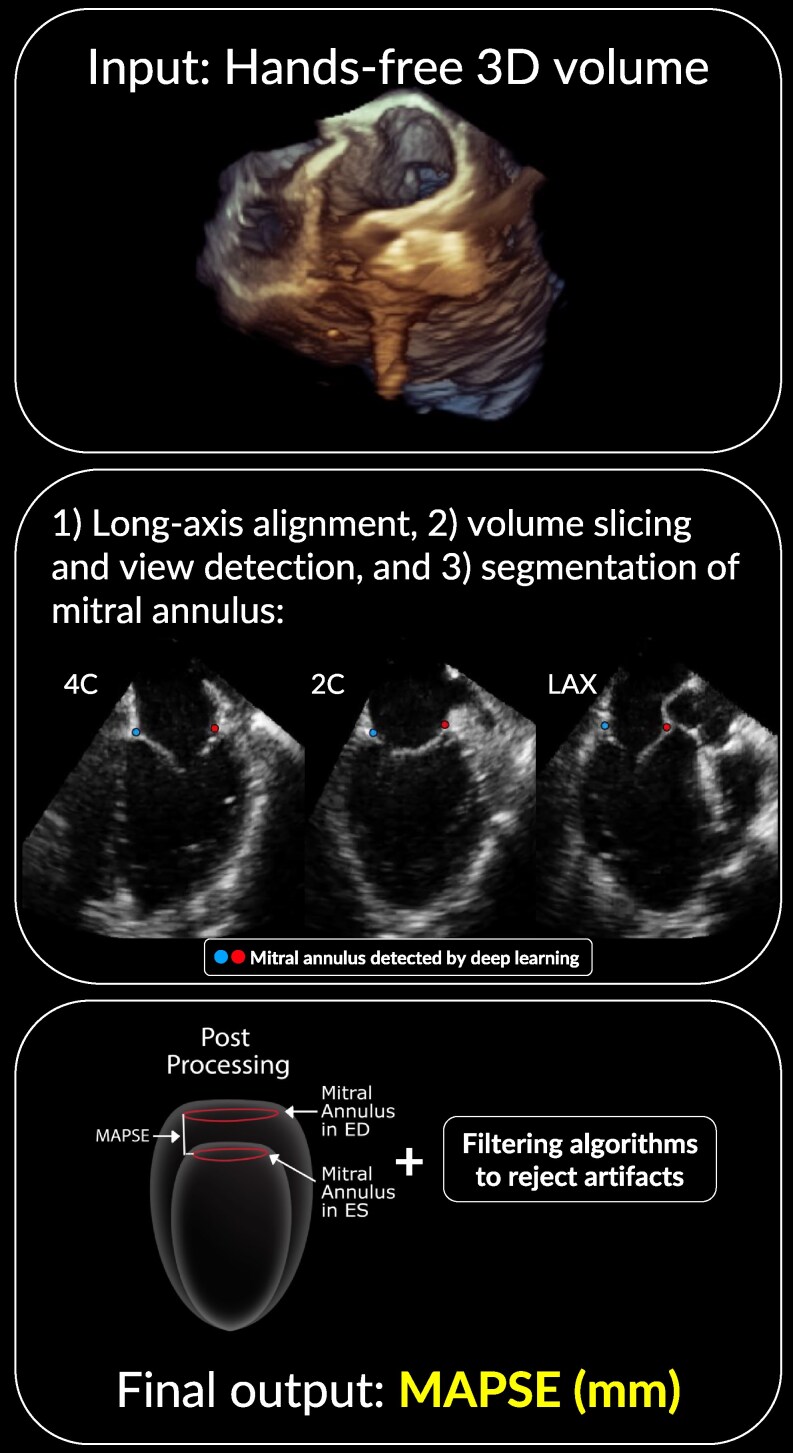
Overview of the pipeline of 3D autoMAPSE. *Top panel*: The input is a single-beat full-volume 3D recording obtained in a hands-free manner. *Middle panel*: Three deep learning networks were tasked for (1) long-axis alignment, (2) volume slicing and view detection, and (3) segmentation of the mitral annulus. Their combined efforts detect the mitral annulus (red and blue points) in reconstructed, standardized midesophageal images. *Bottom panel*: MAPSE is reported after a set of post-processing computations and filtering algorithms to reject artefacts. 3D autoMAPSE, automatic measurements of mitral annular plane systolic excursion by 3D transesophageal echocardiography; MAPSE, mitral annular plane systolic excursion.

The technical methodology of 3D autoMAPSE has been described previously.^7^ Briefly, the term *3D autoMAPSE* comprises the pipeline for automatic estimations of MAPSE from 3D TEE images, which comprises three deep learning convolutional neuronal networks (CNNs) trained in a supervised manner plus a set of post-processing computations to obtain MAPSE from a 3D volume (*Figure 3*). The first deep learning network minimized foreshortening by aligning the 3D left ventricle along its longitudinal axis and predicting the image plane with the maximal LV length. The second network detected the standardized midesophageal two-chamber (2C), four-chamber (4C), and LV long-axis (LAX) views by classifying LAX and extrapolating 2C and 4C by imaging planes at 60 degrees rotations from LAX. These two networks performed once for each TEE recording. The third network, a landmark detection CNN encoder–decoder network, was tasked to perform a discrete 3D segmentation of the mitral annulus.^7^ This segmentation focused on the mitral annulus’ intersections within the detected 2C, 4C, and LAX. The network performed the segmentation by estimating heat maps and detected the mitral annulus in the centre of mass of these maps. To reduce the impact of noisy or missing detections, additional segmentations were performed on all points inside a 15-degree arc on each side of the three standardized views. The centre of mass of these arcs then defined each LV wall from which MAPSE was subsequently estimated. Additional segmentations are computationally demanding, so 15 degrees was arbitrarily chosen to balance the analysis time with the advantages of 3D segmentation. The segmentation was performed for every volume of each 10-cycle recording.

The post-processing computations were designed to isolate the longitudinal excursion of the mitral annulus, filter artefacts in the segmentation, and report the final measurement of 3D MAPSE. To isolate the longitudinal excursion, rotation correction was performed to align the excursion along the LV longitudinal axis.^7^ Next, the artefacts were filtered, first per volume, using algorithms that excluded estimates if the estimated annular point moved more than 5 mm from its preceding volume or was more than 5 mm from its closest neighbours.^7^ Then, another algorithm filtered the entire electrocardiogram-defined cardiac cycle and discarded a cycle if the annulus was detected in less than 60% of the volumes of that cycle. Finally, the measurement of 3D MAPSE was computed as the mean of all feasible sub-measurements obtained from one recording comprising 10 possible cardiac cycles and six possible walls.

Thus, the feasibility of 3D autoMAPSE required all three networks to perform successfully and the filtering algorithms to pass the measurement of MAPSE from at least one cycle and one wall.

### Manual echocardiographic measurement

**Figure 4:**
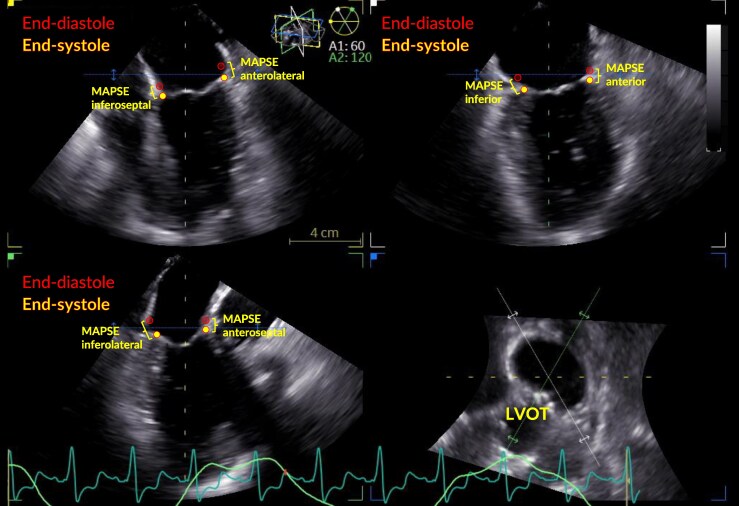
Manual measurement of MAPSE in a multiplanar reconstructed 3D transesophageal image (4D Views®, EchoPAC software version 204, GE Healthcare, Horten, Norway). *Filled points*: The mitral annulus at its lowest position at end-systole. *Open points*: The mitral annulus at its lowest position in a preceding frame. *Brackets*: MAPSE for each wall was measured as the absolute excursion. 3D manual MAPSE was calculated as the mean of all feasible measurements. LVOT, left ventricular outflow tract; MAPSE, mitral annular plane systolic excursion.

We manually measured MAPSE in the 3D volume (3D manual MAPSE) using 4D Views® in EchoPAC (software version 204, GE Healthcare, Horten, Norway) while being blinded to the 3D autoMAPSE measurements. 4D Views® is a manual tool that obtains multiplanar reconstructions of three orthogonal views separated by 60 degrees fixed rotation. Using this tool, we obtained the three standardized midesophageal views by manually reconstructing LAX (*Figure 4*); 2C and 4C followed without additional adjustments. To obtain LAX, the volume was aligned along the LV longitudinal axis and carefully rotated so one of the planes transected the middle of the aortic valve while ensuring that the LV outflow tract was excluded in 4C. Next, MAPSE was measured in all six walls as the absolute annular excursion between the highest and the lowest positions using callipers (*Figure 4*). All 10 cycles were manually measured by one operator, but only in the triplicate recordings. One of these recordings was used to assess interobserver variability by a second operator who repeated the measurements offline on recordings from 20 randomly selected patients while blinded to the previous results.

We also measured 2D autoMAPSE and manual MAPSE, of which the methods and results have been previously described.^11,14^ Additionally, we recorded data during hemodynamic stability to calculate arterial elastance (Ea) and end-systolic elastance (Ees). Ea was calculated as 0.9 times the systolic radial artery pressure divided by the Doppler-estimated stroke volume from the LV outflow tract obtained from either a transgastric LAX view or a deep transgastric view. For this calculation, the LV outflow tract diameter was measured in a 2D midesophageal long-axis view of the aortic valve. Ees was calculated non-invasively using the method described by Chen *et al*.,^17^ where we used LVEF obtained by Simpson’s biplane method of disks, LV ejection times obtained from the R-waves and LV outflow tract Doppler, the stroke volume from the same Doppler signal, and finally, the systolic and diastolic artery pressure obtained from the radial artery. To minimize LV foreshortening, we measured LVEF in simultaneous biplane images obtained using Multi-D® (GE Healthcare, Horten, Norway). Ventriculoarterial coupling was calculated as Ea/Ees ratio. Cardiac output was calculated as the heart rate times stroke volume, and cardiac power output was estimated as cardiac output times the mean arterial pressure divided by 451.^18^ Each of these 2D measurements was the mean of three to five cycles.

### Postoperative cardiac biomarkers

ProBNP and hs-TnT were measured at four time points (*Figure 2C*): (i) during continuous monitoring, (ii) 11 p.m. the same evening, (iii) 6 a.m. of postoperative Day 1, and (iv) 11 p.m. on postoperative Day 1. Both biomarkers were analysed immunologically using Cobas 8000 (Roche Diagnostics, Norway). The within-lab coefficient of variation was 2.9% for ProBNP and 4.0% for hs-TnT.

### Statistical analysis

We report each *measurement* of MAPSE as the mean of all feasible sub-measurements obtained from one recording which comprised 10 cardiac cycles and 6 walls. The bias, limits of agreement (LOA), and precision were only assessed in the triplicate recordings using the linked replicated model described by Carstensen,^14,19^ which adjusts the Bland–Altman analysis for repeated measurements. From this model, the precision of 3D autoMAPSE and manual MAPSE was calculated using the residual standard deviations of each respective method and reported as the least significant change (LSC) using the following formula^19,20^:


$$LSC=\sqrt{2}\times 1.96\;\times \frac{Standard\;deviation}{\sqrt{Number\;of\;measurements}}.$$

Using the conventional Bland–Altman analysis,^21^ the interobserver variability of 3D manual MAPSE was assessed between two observers measuring the same recording.

Additionally, 3D autoMAPSE was compared with manual measurements of LVEF, non-invasive hemodynamics, Ea/Ees ratio, and peak postoperative cardiac biomarkers using the nonparametric correlation coefficient Spearman’s *rho*.

To explore the potential value of continuous monitoring using 3D autoMAPSE, we compared the time-weighted average of continuous 3D autoMAPSE measurements with single echocardiographic measurements for predicting peak postoperative biomarkers. For this analysis, the time-weighted average was first obtained by integrating the continuous measurements over time and dividing them by the total minutes monitored. Thus, the term *continuous 3D autoMAPSE* refers to the time-weighted average of the continuously obtained measurements, whereas *single 3D autoMAPSE* is the measurement obtained at the time point of the test-retest protocol. Next, Spearman’s *rho* between echocardiographic measurements and biomarkers were obtained, and the correlation coefficients were compared.

Data are reported as the mean ± standard deviation or median [25–75 percentiles]. The statistical analysis plan was established *a priori*. Missing data were not replaced. The sample size was decided based on our experience with similar method comparison studies. Statistical significance was defined as a *P* < 0.05. Stata 18.0 (StataCorp LLC) was used for all the analyses.

## Results

Fifty patients were included after on-pump cardiac surgery (*Table 1*). Of these, 24 (48%) underwent isolated coronary artery bypass grafting, 20 (40%) had aortic valve replacement, 4 (8%) had mitral valve replacement, 1 (2%) had mitral valve repair, and 6 (12%) underwent surgery on the thoracic aorta; 9 (18%) of these 50 patients had combined procedures. Aortic cross-clamp time was 72 ± 32 min, and cardiopulmonary bypass time was 97 ± 45 min. Preoperative hs-TnT was 17 [11–61] ng/L, and preoperative ProBNP was 490 [141–1387] ng/L.

**Table 1:** Patient characteristics (N = 50)

Preoperative characteristics	Value
Age (years)	67 ± 8
Male (*n*, %)	38 (76)
Body surface area (m^2^)	1.98 ± 0.2
EuroScore II	2.6 ± 2.7
Myocardial infarction within 90 days (*n*, %)	15 (30)
Preoperative LVEF (%)	47 (12)
Preoperative LVEF < 30% (*n*, %)	4 (8)
NYHA class 1 (*n*, %)	22 (44)
NYHA class 2 (*n*, %)	14 (28)
NYHA class 3 (*n*, %)	14 (28)
NYHA class 4 (*n*, %)	0 (0)
Mild mitral regurgitation (*n*, %)	28 (56)
Moderate mitral regurgitation (*n*, %)	1 (2)
Severe mitral regurgitation (*n*, %)	5 (10)
Diabetes mellitus on insulin (*n*, %)	1 (2)
Creatinine clearance < 85 mL/min (*n*, %)	20 (40)
Atrial fibrillation (*n*, %)	3 (6%)
Echocardiographic measurements in the ICU	
3D autoMAPSE (mm)	5.2 ± 1.7
3D manual MAPSE (mm)	6.6 ± 2.0
2D autoMAPSE (mm)	6.9 ± 2.0
2D manual MAPSE (mm)	7.6 ± 2.0
LVEF (%)	50 ± 15
LV end-diastolic volume (mL)	119 ± 43
LV end-diastolic volume index (mL/m^2^)	59 ± 20
LV end-systolic volume (mL)	63 ± 41
LV end-systolic volume index (mL/m^2^)	31 ± 19
Ea/Ees ratio	1.33 ± 0.6
Continuously monitored variables in the ICU	
Propofol (mg/kg/h)	2.4 [2.0–2.9]
Norepinephrine (mcg/kg/min)	0.03 [0.02–0.07]
Mean arterial pressure (mmHg)	77 ± 7
Heart rate (bpm)	75 ± 15
Central venous pressure (mmHg)	7 ± 3
Lactate (mmol/L)	1.0 ± 0.4
ScvO_2_ (%)	67 ± 6
PcvaCO_2_ (kPa)	1.0 ± 0.2

MAPSE values are the mean of all available walls and cardiac cycles obtained from the triplicate recordings. Data are given as *n* (%), mean ± SD, and median [25–75 percentiles].

2D autoMAPSE, automatic measurements of mitral annular plane systolic excursion by 2D transesophageal echocardiography; 3D autoMAPSE, automatic measurements of mitral annular plane systolic excursion by 3D transesophageal echocardiography; Ea, effective arterial elastance; Ees, end-systolic elastance; ICU, intensive care unit; LV, left ventricular; LVEF, left ventricular ejection fraction, MAPSE, mitral annular plane systolic excursion; NYHA, New York Heart Association; PcvaCO_2_, central veno-arterial CO_2_ gap; ScvO_2_, central venous oxygen saturation.

Overall, 3D measurements of MAPSE were numerically lower than the previously published 2D measurements (*Table 1*). 3D autoMAPSE ranged from 1.0 to 10.7 mm, and 3D manual MAPSE ranged from 2.7 to 11.0 mm, whereas 2D autoMAPSE ranged from 2.3 to 11.1 mm, and 2D manual MAPSE ranged from 2.9 to 12.5 mm. The temporal resolution was 19.4 ± 2.8 volumes per second for the 3D measurements and 48 ± 7 frames per second for the 2D measurements.

### Feasibility and precision

3D autoMAPSE had excellent feasibility (99%), providing measurements in 1224 of 1232 total recordings during continuous monitoring. One measurement took less than a minute (55 ± 15 s). Most of the feasible recordings (1074 of 1224; 88%) contained measurements from at least four walls, and more than half (705 of 1224; 58%) contained all six, averaging at 5.1 ± 1.1 walls per recording. A sub-group analysis of each wall showed that the anterior wall had the highest feasibility of 95% (*Table 2*). 3D autoMAPSE also had excellent feasibility in the five patients undergoing mitral valve surgery (110 of 111 recordings; 99%). Only 14 (1.1%) unscheduled probe adjustments were required, mainly due to spontaneous or nursing-related patient movement; the maximum number of unscheduled adjustments per patient was 2. No recordings were excluded due to poor image quality, arrhythmias, pacing, or mitral annular calcifications. One patient was monitored for 100 min due to early extubation, and another patient was monitored for 50 min due to the need for emergent reoperation. No patients suffered complications from continuous TEE.

**Table 2:** Sub-group analysis of comparing 3D MAPSE by each individual wall

Wall	Feasibility of 3D autoMAPSE (%)	3D autoMAPSE (mm)	3D manual MAPSE (mm)	Bias (95% LOA)
Anterior	95	4.9 ± 2.0	6.2 ± 1.8	−1.4 (−5.2 to 2.5)
Inferior	80	5.6 ± 2.0	6.5 ± 2.2	−0.9 (−4.5 to 2.8)
Anterolateral	82	6.1 ± 2.5	8.7 ± 3.0	−3.0 (−8.1 to 2.1)
Inferoseptal	81	5.4 ± 2.2	6.3 ± 2.4	−1.0 (−5.2 to 3.2)
Anteroseptal	93	4.6 ± 1.8	5.3 ± 1.4	−0.7 (−4.0 to 2.5)
Inferolateral	83	6.2 ± 2.6	7.0 ± 3.0	−0.7 (−5.9 to 4.5)

3D autoMAPSE, automatic measurements of mitral annular plane systolic excursion by 3D transesophageal echocardiography; LOA, limits of agreement; MAPSE, mitral annular plane systolic excursion.

**Figure 5:**
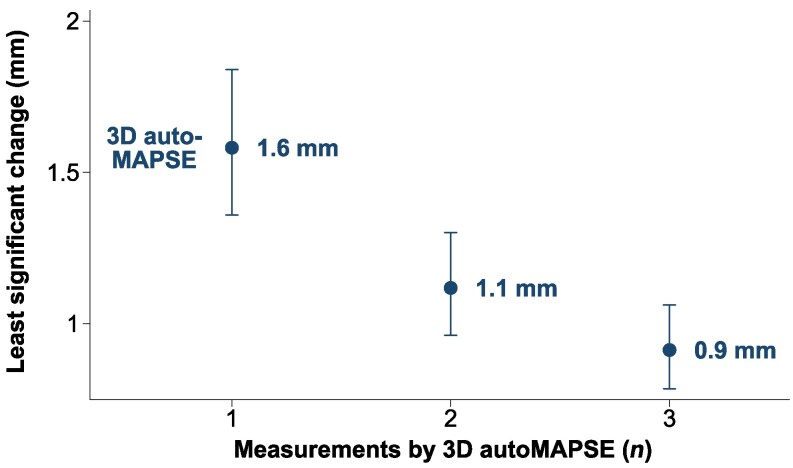
The mean precision of 3D autoMAPSE in all 50 patients, reported as the LSC. Each measurement (*n*) is the mean MAPSE obtained from one recording comprising up to 10 cardiac cycles and 6 walls. 3D autoMAPSE, automatic measurements of mitral annular plane systolic excursion by 3D transesophageal echocardiography.

The highly feasible measurements were also precise, where the LSC indicates that one measurement by 3D autoMAPSE (i.e. the mean MAPSE of up to 10 cardiac cycles and 6 walls) could detect changes of 1.6 mm, and the mean of three such measurements could detect changes as small as 0.9 mm (*Figure 5*).

### Clinical validation

**Figure 6:**
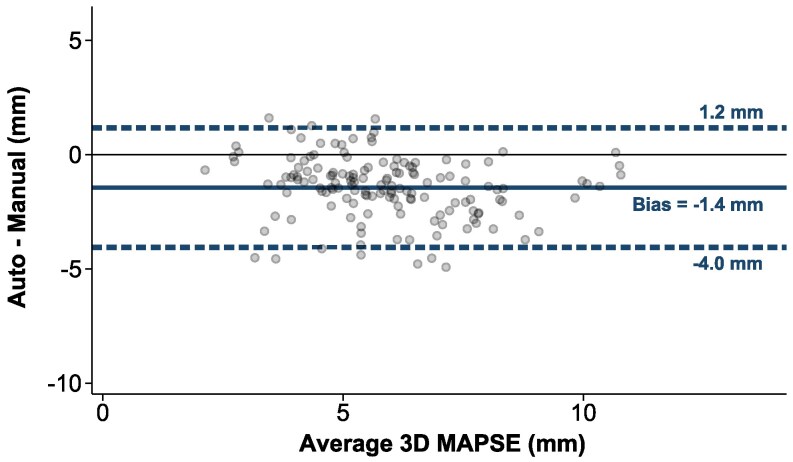
Bland–Altman plot comparing 3D autoMAPSE with 3D manual MAPSE. Each data point is the mean MAPSE of all feasible sub-measurements obtained from one recording which comprised 10 cardiac cycles and 6 walls. One patient had missing triplicate images, in which we measured MAPSE manually in the last image of the protocol, yielding a total of 148 data points. 3D autoMAPSE, automatic measurements of mitral annular plane systolic excursion by 3D transesophageal echocardiography; MAPSE, mitral annular plane systolic excursion.

3D autoMAPSE had narrow limits of agreement with 3D manual MAPSE, but the bias was significant at −1.4 mm (*Figure 6*). This bias was not fully explained by errors in 3D autoMAPSE segmentation, illustrated by an example patient where the bias was −1.6 mm despite excellent detection of the mitral annulus (Supplementary data online, *Video S1*).

A sub-group analysis of the individual walls revealed that all walls had a negative bias, where the largest bias of −3.0 mm was found in the anterolateral wall (*Table 2*). However, excluding this wall did not improve the overall bias or agreement by much (bias −1.1 mm; LOA −3.6 to 1.4 mm).

Another sub-group analysis for the five patients who underwent mitral valve surgery showed that the agreement between 3D autoMAPSE and 3D manual MAPSE was comparable with the global cohort (bias −0.6 mm; LOA −3.4 to 2.2 mm). However, the method was less precise in this sub-group, where the LSC was 2.4 mm for one measurement, 1.7 mm for the mean of two measurements, and 1.4 mm for the mean of three.

The LSC of 3D manual MAPSE was 0.9 mm for one measurement, indicating that the reference method had excellent precision. Likewise, this method had excellent interobserver variability (bias 0.3 mm, LOA −1.2 to 1.9 mm).

Validation of 3D autoMAPSE against 2D echocardiographic measurements shows significant correlation with other important hemodynamic parameters and strongest so for Ea/Ees ratio (*Table 3*).

**Table 3:** Spearman’s *rho* between 3D autoMAPSE and 2D echocardiographic measurements

2D echocardiographic parameter	Observations (*n*)	Spearman’s *rho*	*P*-value
Cardiac output	44	0.38	0.011
Cardiac power output	44	0.40	0.007
Ea	44	−0.21	0.170
Ea/Ees ratio	41	−0.48	0.002
Ees	41	0.35	0.026
LVEF	42	0.41	0.008
Stroke volume	44	0.35	0.020
Velocity–time integral	44	0.24	0.121

3D autoMAPSE, automatic measurements of mitral annular plane systolic excursion by 3D transesophageal echocardiography; Ea, effective arterial elastance; Ees, end-systolic elastance; LVEF, left ventricular ejection fraction.

Continuous 3D autoMAPSE was significantly associated with both ProBNP and hs-TnT, although the association was somewhat weak (*Table 4*). However, this association was slightly stronger than the association between biomarkers and the more conventional LVEF (*Table 4*). Furthermore, any of the single echocardiographic measurements were at best only significant for ProBNP and not hs-TnT (*Table 4*). Peak values of postoperative hs-TnT were 376 [226–580] ng/L, and ProBNP was 1530 [1031–3909] ng/L. Overall, hs-TnT peaked at 11 p.m. on the day of surgery, while ProBNP peaked at 11 p.m. on postoperative Day 1.

**Table 4:** Spearman’s *rho* between echocardiographic measurements and postoperative cardiac biomarkers

Echocardiographic measurement	Peak postoperative ProBNP	*P*-value (ProBNP)	Peak postoperative hs-TnT	*P*-value (hs-TnT)
Continuous 3D autoMAPSE	−0.37	0.008^a^	−0.28	0.047^a^
Single 3D autoMAPSE	−0.29	0.041^a^	−0.19	0.192
3D manual MAPSE	−0.38	0.007^a^	−0.12	0.391
2D manual MAPSE	−0.34	0.026^a^	−0.24	0.116
LVEF	−0.31	0.046^a^	−0.16	0.326
Ea/Ees ratio	0.26	0.107	0.06	0.711
Ees	−0.23	0.142	−0.02	0.910
Velocity–time integral	−0.08	0.593	−0.10	0.494
Stroke volume	−0.06	0.715	−0.18	0.243
Cardiac output	−0.00	0.990	−0.19	0.206

*Continuous 3D autoMAPSE* refers to the time-weighted average of measurements obtained every 5 min for 2 h. *Single 3D autoMAPSE* refers to the measurement obtained at the time point of the test-retest protocol. All other echocardiographic measurements are single measurements.

3D autoMAPSE, automatic measurements of mitral annular plane systolic excursion by 3D transesophageal echocardiography; MAPSE, mitral annular plane systolic excursion; Ea, effective arterial elastance; Ees, end-systolic elastance; hs-TnT, high-sensitivity troponin-T; LVEF, left ventricular ejection fraction; MAPSE, mitral annular plane systolic excursion; ProBNP, N-terminal pro B-type natriuretic peptide.

^a^Statistical significance.

**Figure 7:**
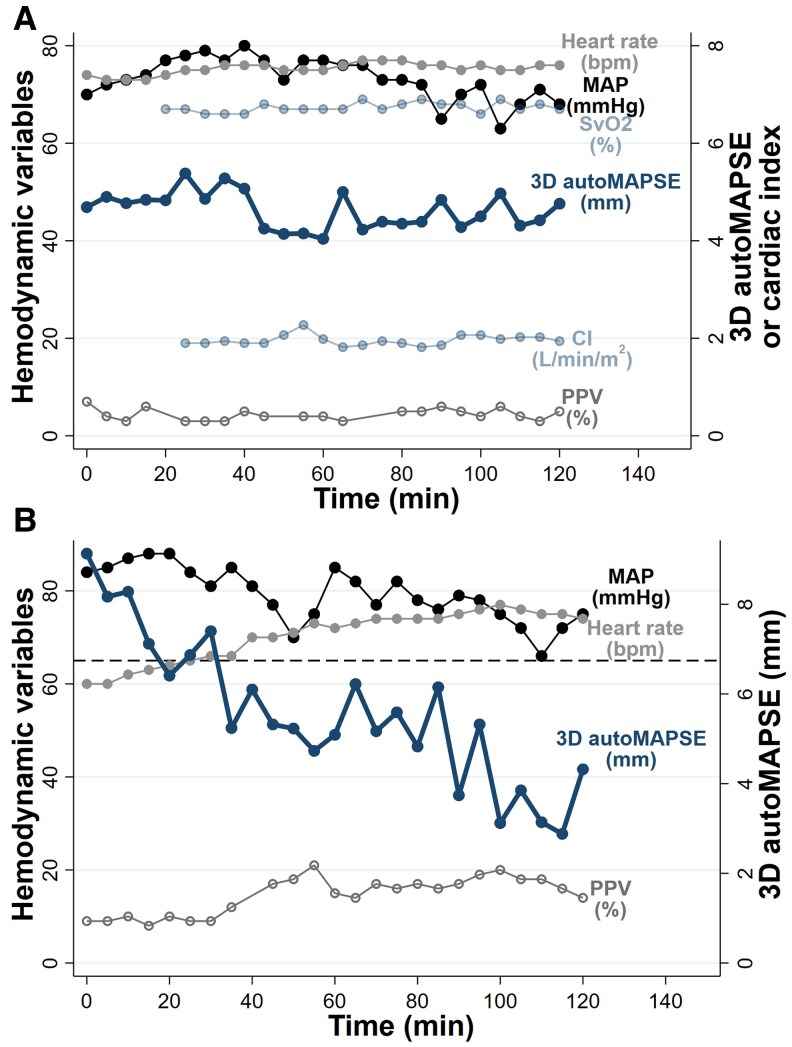
Two case examples showing continuous measurements of 3D autoMAPSE. (*A*) An example case showing a hemodynamically stable patient. This patient was monitored by routine equipment, 3D autoMAPSE, and a pulmonary artery catheter. All monitored parameters were stable, including 3D autoMAPSE. (*B*) An example case showing a patient where an unexpected deterioration of left ventricular function was detected by 3D autoMAPSE. The clinical picture for this patient was relatively unremarkable, except for a slight increase in norepinephrine (0.03–0.07 mcg/kg/min). However, 3D autoMAPSE revealed a decrease from 9.1 to 2.9 mm. This decrease was larger than the method’s LSC (1.6 mm) and therefore likely real. This case demonstrates that continuous monitoring using 3D autoMAPSE can detect unexpected deteriorations in LV function. 3D autoMAPSE, automatic measurements of mitral annular plane systolic excursion by 3D transesophageal echocardiography; CI, cardiac index; MAP, mean arterial pressure; PPV, pulse pressure variability; SvO_2_, mixed venous oxygen saturation.

To illustrate the clinical potential, we provide two example cases from our data (*Figure 7*). The first example shows a hemodynamically stable patient (*Figure 7A*). The second shows a patient with deterioration in LV function (*Figure 7B*). This deterioration was unexpected by conventional monitoring but rather obvious by 3D autoMAPSE (*Figure 7B*). The postoperative drains registered 320 mL of bleeding during the first 2 h, and the associated increase in pulse pressure variation suggests that a decrease in preload caused the deterioration in MAPSE, a phenomenon we have demonstrated previously using 2D autoMAPSE.^12^

## Discussion

Our findings show that continuous monitoring by 3D autoMAPSE had excellent feasibility in perioperative patients. The highly feasible measurements were precise and had adequate agreement with manual echocardiography and postoperative cardiac biomarkers. Thus, 3D autoMAPSE provides continuous monitoring of LV function and can potentially aid cardiopulmonary management.

### Implications for clinical practice and research

Continuous monitoring of LV function by 3D autoMAPSE may potentially benefit both clinical practice and research. We foresee at least two applications for clinical practice. The first resembles the current practice of point-of-care echocardiography where the clinician actively integrates all clinical information at the bedside. Here, 3D autoMAPSE can provide quick and effortless quantification of LV function, thereby aiding in assessing the patient’s overall state. This application is likely most useful during high-risk situations or during frequent therapeutic adjustments. In these settings, the clinician can monitor MAPSE continuously by adjusting the imaging frequency. The second application is to use 3D autoMAPSE for passive continuous monitoring while the clinician attends to other matters. This application utilizes frequent and precise measurements for earlier detection and treatment of LV dysfunction. Our experience suggests that this latter application is most feasible when the patients are immobilized, such as during surgery, percutaneous interventions, or in the early phase of critical illness.

Clinical interpretation of 3D MAPSE should be done by incorporating its changes together with other available hemodynamic variables. We caution against interpreting changes in MAPSE as changes in contractility because MAPSE is a load-dependent parameter of global LV function, just like LVEF and global longitudinal strain. However, we do not consider the load dependency as a limitation *per se*, because cardiac load is important for myocardial energetics.^22^ Thus, monitoring load-dependent parameters of LV function is still important for myocardial stress, myocardial injury, and cardiac complications in perioperative patients. In this regard, we found that 3D autoMAPSE was significantly correlated with Ea/Ees ratio (reflecting mechanical myocardial energetics), ProBNP (reflecting myocardial stress), and hs-TnT (reflecting myocardial injury). Additionally, we emphasize that previous studies demonstrated that changes in MAPSE from each individual wall reflect changes in global LV function and not changes in regional function of that wall.^23–26^ Thus, 3D autoMAPSE cannot be used to reliably detect regional myocardial ischaemia unless global LV function is also affected.

For research, continuous monitoring by 3D autoMAPSE may provide more detailed data on the temporal dynamics of LV function compared with current methods in echocardiography. Such high-resolution data can aid in studying the temporal interactions between LV function and clinical interventions or other hemodynamic variables. The time-weighted average from continuous monitoring is also less affected by short-lived changes in LV function that are clinically insignificant. Indeed, insignificant changes in LV function, such as the ones seen during changes in the patients’ positioning,^12^ may explain why single echocardiographic measurements had a weaker association with postoperative hs-TnT compared with the time-weighted average from continuous monitoring (*Table 4*). Thus, continuous 3D autoMAPSE can potentially provide valuable data on LV function in hemodynamically unstable patients.

### Technological considerations

3D autoMAPSE overcomes two obstacles that have previously limited echocardiography for continuous monitoring. Although manual echocardiography is invaluable for diagnosing LV dysfunction during cardiopulmonary failure, this current method will always be intermittent because the time-consuming image acquisition and manual measurements required for continuous quantitative monitoring occupy the clinician from more important tasks. Furthermore, those measurements may fail to detect subtle but important changes in LV function because they lack precision.^27^ The common practice of qualitative assessments further reduces precision by categorizing quantitative data on LV function. Intermittent and semi-qualitative assessments may have contributed to the negative results of a recent randomized trial where a prolonged indwelling TEE probe was used to guide therapy.^28^

These issues have motivated the use of deep learning for automated image analysis. However, although deep learning reduces analysis time and improves precision,^29–31^ most methods are still ineffective for continuous monitoring because they are limited to transthoracic echocardiography. This is why we have applied deep learning to TEE. Our previous attempt showed that 2D autoMAPSE was promising for continuous hands-free imaging,^14^ but 2D imaging planes are vulnerable to even small probe dislocations. This problem is partly solved by 3D autoMAPSE because it overcomes the need for fixed imaging planes. Additionally, 3D autoMAPSE improved the precision further (LSC 1.6 vs. 2.4 mm by hands-free 2D autoMAPSE^14^) by averaging MAPSE from up to six walls. Better precision is essential for monitoring because it reduces the threshold to distinguish actual changes from measurement errors.^19,20^ Thus, two of echocardiography’s longstanding obstacles for continuous monitoring of LV function, the time-consuming image acquisitions and imprecise manual measurements, are partly overcome by 3D autoMAPSE.

Several aspects of our study suggest that our findings on 3D autoMAPSE may be generalizable to other more high-risk populations. First, our perioperative patients under sedation and mechanical ventilation differ significantly from the training data used for 3D autoMAPSE, which comprised awake cardiology patients presenting for diagnostic TEE.^7^ Second, none of our patients were excluded due to poor image quality. The image quality is generally poor in single-beat full-volume 3D images and may be further worsened by postoperative factors such as tissue disruption and dried mucosa from prolonged scanning time. Despite these issues, 3D autoMAPSE showed excellent feasibility and precision, suggesting that our findings may be generalizable to other populations.

Still, several technical issues remain. First, the measurements of 3D autoMAPSE had a small, albeit significant bias (−1.4 mm). As our example case (Supplementary data online, *Video S1*) suggests, this bias is not fully explained by erroneous segmentation. Instead, the main explanation is presumably that 3D autoMAPSE reports the isolated longitudinal excursion of the mitral annulus after correcting any motion that is not along the LV longitudinal axis. This correction was not done with manual MAPSE because we considered it difficult to standardize and its importance uncertain. Thus, manual MAPSE measures the absolute excursion of the mitral annulus, which may be slightly larger than the isolated longitudinal component. This explanation is also concordant with the observed bias between 2D autoMAPSE and manual MAPSE (*Table 1*). However, the clinical importance of this bias is unclear because the goal of continuous monitoring is to detect *relative* changes, not absolute values.^20^

Another issue when comparing 3D autoMAPSE with manual measurements is the inaccuracies and variability inherent to human measurements. These inaccuracies can render the correlation and Bland–Altman analysis difficult to interpret when the results show only moderate agreement. Thus, a comparison of 3D autoMAPSE with established and prognostically important cardiac biomarkers served as a complementary approach to assess whether 3D autoMAPSE in fact reflected myocardial dysfunction independent of manual echocardiographic measurements.

A more important issue is the lower absolute values of 3D autoMAPSE, which are mainly due to the lower temporal resolution of the single-beat full-volume 3D images compared with 2D images. Additionally, the isolated longitudinal component measured by 3D autoMAPSE is also angle-independent, which further contributes to lower MAPSE values compared with the angle-dependent M-mode MAPSE; indeed, the angle dependency of M-mode increases MAPSE by a factor of one over the cosine of the angle of deviation, a phenomenon also seen when comparing MAPSE by M-mode vs. speckle tracking.^32,33^ Finally, as shown in the present study as well as others,^12,14,34^ MAPSE is lower in the septal segments than in the lateral ones. Therefore, future clinicians utilizing 3D autoMAPSE monitoring must keep in mind which walls the final MAPSE measurement is based on, since global 3D MAPSE comprising several septal segments may be lower than MAPSE values obtained from the lateral walls only. Although an LSC of 1.6 mm suggests excellent precision, the low 3D autoMAPSE values can potentially have serious implications because they may render the *relative* precision inadequate. The adequacy of LOA is also affected, but this is less important because the reference method was manual echocardiographic measurements, not a gold standard, and because the relative changes of interest are independent of agreement. This issue can be resolved by assessing the ability of 3D autoMAPSE to track changes in LV function during hemodynamic interventions and comparing those changes with different reference methods. However, the present study was not designed for this purpose.

### Limitations

Our study has some additional limitations. First, this study did not assess real-time monitoring by 3D autoMAPSE because our protocol was designed to prioritize the real-time aspect of 2D autoMAPSE in the same patients.^11,14^ Second, TEE may cause complications even if none occurred in our patients. Although severe complications of TEE occur very rarely, recent reports suggest that TEE complications may occur more frequently than previously assumed.^35,36^ Therefore, it is imperative not to override the manufacturer’s safety limits regarding probe temperature. Future use of smaller TEE probes may improve safety. Third, the scheduled probe adjustment may inflate the reported feasibility compared with a completely hands-free system. Fourth, our sample size is small, which also limits the statistical power for a multivariable analysis in predicting postoperative biomarkers. Although both ProBNP and hs-TnT have shown prognostic value in patients after cardiac surgery,^5,6^ they are unspecific and may not imply acute heart failure or myocardial infarction early after cardiac surgery. Future studies should address the ability to predict more patient-centred outcomes. Fifth, our study aimed to test the feasibility and validate 3D autoMAPSE as a tool for continuous monitoring. However, the ideal population for continuous 3D autoMAPSE may not be patients scheduled for cardiac surgery, but critically ill patients with more severe cardiopulmonary failure. Future studies should address the use of continuous 3D autoMAPSE in these higher-risk patients. Sixth, MAPSE measures the annular excursion without the need to visualize the rest of the left ventricle because the LV apex is usually stationary. This feature is usually an advantage but may render MAPSE inaccurate in situations where the apex is non-stationary, such as large pericardial effusions. Seventh, 3D autoMAPSE requires training and extended use of state-of-the-art equipment, which challenges the availability of medical resources. Thus, selecting the appropriate patients for 3D autoMAPSE monitoring is a mandatory priority. Finally, this study has not addressed the impact of 3D autoMAPSE on therapies.

## Conclusion

Continuous monitoring of LV function in perioperative patients was highly feasible by 3D autoMAPSE. The measurements were precise and in agreement with manual echocardiography and postoperative cardiac biomarkers. Thus, continuous 3D autoMAPSE may be an attractive complement to hemodynamic monitoring that can aid cardiopulmonary management.

## Supplementary Material

qyaf052_Supplementary_Data

## Data Availability

The datasets used and analyzed during the current study cannot be shared due to a lack of patient consent for this purpose.

## Lead author biography

Jinyang Yu (b. 1992), MD, PhD, graduated from the University of Tromsø, Norway, in 2017. His previous research aims to develop continuous monitoring of cardiac function in intensive care patients by utilizing deep learning methods for automated analysis of transesophageal echocardiographic images. His clinical work is currently as an anaesthesiology fellow at St. Olav’s University Hospital, Trondheim, Norway, where he pursues his interests in cardiopulmonary monitoring and pathophysiology.

## References

[1] Flu WJ, van Kuijk JP, Hoeks SE, Kuiper R, Schouten O, Goei D et al Prognostic implications of asymptomatic left ventricular dysfunction in patients undergoing vascular surgery. Anesthesiology 2010;112:1316–24. 20502115 10.1097/ALN.0b013e3181da89ca

[2] Ternacle J, Berry M, Alonso E, Kloeckner M, Couetil J-P, Dubois Randé J-L et al Incremental value of global longitudinal strain for predicting early outcome after cardiac surgery. Eur Heart J—Cardiovasc Imaging 2013;14:77–84. 22893712 10.1093/ehjci/jes156

[3] London MJ, Tubau JF, Wong MG, Layug E, Hollenberg M, Krupski WC et al The “natural history” of segmental wall motion abnormalities in patients undergoing noncardiac surgery. Anesthesiology 1990;73:644–55. 2221433 10.1097/00000542-199010000-00010

[4] Cecconi M, De Backer D, Antonelli M, Beale R, Bakker J, Hofer C et al Consensus on circulatory shock and hemodynamic monitoring. Task force of the European society of intensive care medicine. Intensive Care Med 2014;40:1795–815. 25392034 10.1007/s00134-014-3525-z PMC4239778

[5] Mauermann E, Bolliger D, Fassl J, Grapow M, Seeberger EE, Seeberger MD et al Postoperative high-sensitivity troponin and its association with 30-day and 12-month, all-cause mortality in patients undergoing on-pump cardiac surgery. Anesth Analg 2017;125:1110–7. 28537984 10.1213/ANE.0000000000002023

[6] Mauermann E, Bolliger D, Fassl J, Grapow M, Seeberger EE, Seeberger MD et al Absolute postoperative B-type natriuretic peptide concentrations, but not their general trend, are associated with 12-month, all-cause mortality after on-pump cardiac surgery. Anesth Analg 2017;125:753–61. 28753169 10.1213/ANE.0000000000002291

[7] Taskén AA, Berg EAR, Grenne B, Holte E, Dalen H, Stølen S et al Automated estimation of mitral annular plane systolic excursion by artificial intelligence from 3D ultrasound recordings. Artif Intell Med 2023;144:102646. 37783546 10.1016/j.artmed.2023.102646

[8] Hu K, Liu D, Herrmann S, Niemann M, Gaudron PD, Voelker W et al Clinical implication of mitral annular plane systolic excursion for patients with cardiovascular disease. Eur Heart J—Cardiovasc Imaging 2013;14:205–12. 23161791 10.1093/ehjci/jes240

[9] Huang SJ, Ting I, Huang AM, Slama M, McLean AS. Longitudinal wall fractional shortening: an M-mode index based on mitral annular plane systolic excursion (MAPSE) that correlates and predicts left ventricular longitudinal strain (LVLS) in intensive care patients. Crit Care 2017;21:292. 29178915 10.1186/s13054-017-1876-x PMC5702151

[10] Bergenzaun L, Öhlin H, Gudmundsson P, Willenheimer R, Chew MS. Mitral annular plane systolic excursion (MAPSE) in shock: a valuable echocardiographic parameter in intensive care patients. Cardiovasc Ultrasound 2013;11:16. 23718803 10.1186/1476-7120-11-16 PMC3679845

[11] Taskén AA, Yu J, Berg EAR, Grenne B, Holte E, Dalen H et al Automatic detection and tracking of anatomical landmarks in transesophageal echocardiography for quantification of left ventricular function. Ultrasound Med Biol 2024;50:797–804. 38485534 10.1016/j.ultrasmedbio.2024.01.017

[12] Yu J, Taskén AA, Flade HM, Skogvoll E, Berg EAR, Grenne B et al Automatic assessment of left ventricular function for hemodynamic monitoring using artificial intelligence and transesophageal echocardiography. J Clin Monit Comput 2024;38:281–91. 38280975 10.1007/s10877-023-01118-x

[13] Berg EAR, Taskén AA, Nordal T, Grenne B, Espeland T, Kirkeby-Garstad I et al Fully automatic estimation of global left ventricular systolic function using deep learning in transoesophageal echocardiography. Eur Heart J—Imaging Methods Pract 2023;1:qyad007. 39044786 10.1093/ehjimp/qyad007 PMC11195714

[14] Yu J, Taskén AA, Berg EAR, Tannvik TD, Slagsvold KH, Kirkeby-Garstad I et al Continuous monitoring of left ventricular function in postoperative intensive care patients using artificial intelligence and transesophageal echocardiography. Intensive Care Med Exp 2024;12:54. 38856861 10.1186/s40635-024-00640-9 PMC11164841

[15] Hahn RT, Abraham T, Adams MS, Bruce CJ, Glas KE, Lang RM et al Guidelines for performing a comprehensive transesophageal echocardiographic examination: recommendations from the American society of echocardiography and the society of cardiovascular anesthesiologists. Anesth Analg 2014;118:21–68. 24356157 10.1213/ANE.0000000000000016

[16] von Elm E, Altman DG, Egger M, Pocock SJ, Gøtzsche PC, Vandenbroucke JP. Strengthening the reporting of observational studies in epidemiology (STROBE) statement: guidelines for reporting observational studies. BMJ 2007;335:806–8. 17947786 10.1136/bmj.39335.541782.AD PMC2034723

[17] Chen CH, Fetics B, Nevo E, Rochitte CE, Chiou KR, Ding PA et al Noninvasive single-beat determination of left ventricular end-systolic elastance in humans. J Am Coll Cardiol 2001;38:2028–34. 11738311 10.1016/s0735-1097(01)01651-5

[18] Fortuni F, Zilio F, Iannopollo G, Ciliberti G, Trambaiolo P, Ceriello L et al Management of temporary mechanical circulatory support devices in cath-lab and cardiac intensive care unit. Eur Heart J—Imaging Methods Pract 2023;1:qyad011. 39044800 10.1093/ehjimp/qyad011 PMC11195697

[19] Carstensen B . Comparing Clinical Measurement Methods: A Practical Guide. Chichester: John Wiley & Sons, Ltd; 2010.

[20] Cecconi M, Rhodes A, Poloniecki J, Rocca GD, Grounds RM. Bench-to-bedside review: the importance of the precision of the reference technique in method comparison studies—with specific reference to the measurement of cardiac output. Crit Care 2009;13:201. 19183431 10.1186/cc7129 PMC2688094

[21] Bland JM, Altman D. Statistical methods for assessing agreement between two methods of clinical measurement. The Lancet 1986;327:307–10. 2868172

[22] Suga H . Ventricular energetics. Physiol Rev 1990;70:247–77. 2181496 10.1152/physrev.1990.70.2.247

[23] Støylen A, Skjærpe T. Systolic long axis function of the left ventricle. Global and regional information. Scand Cardiovasc J 2003;37:253–8. 14534065 10.1080/14017430310015000

[24] Pahlm U, Seemann F, Engblom H, Gyllenhammar T, Halvorsen S, Hansen HS et al Longitudinal left ventricular function is globally depressed within a week of STEMI. Clin Physiol Funct Imaging 2018;38:1029–37. 10.1111/cpf.12521 29701310

[25] Pahlm U, Ostenfeld E, Seemann F, Engblom H, Erlinge D, Heiberg E et al Evolution of left ventricular function among subjects with ST-elevation myocardial infarction after percutaneous coronary intervention. BMC Cardiovasc Disord 2020;20:309. 32600336 10.1186/s12872-020-01540-y PMC7322852

[26] Berg J, Jablonowski R, Nordlund D, Kopic S, Bidhult S, Xanthis CG et al Decreased atrioventricular plane displacement after acute myocardial infarction yields a concomitant decrease in stroke volume. J Appl Physiol 2020;128:252–63. 31854250 10.1152/japplphysiol.00480.2019 PMC7052588

[27] Jozwiak M, Mercado P, Teboul JL, Benmalek A, Gimenez J, Dépret F et al What is the lowest change in cardiac output that transthoracic echocardiography can detect? Crit Care 2019;23:116. 30971307 10.1186/s13054-019-2413-x PMC6458708

[28] Merz TM, Cioccari L, Frey PM, Bloch A, Berger D, Zante B et al Continual hemodynamic monitoring with a single-use transesophageal echocardiography probe in critically ill patients with shock: a randomized controlled clinical trial. Intensive Care Med 2019;45:1093–102. 31273416 10.1007/s00134-019-05670-6

[29] Salte IM, Østvik A, Olaisen SH, Karlsen S, Dahlslett T, Smistad E et al Deep learning for improved precision and reproducibility of left ventricular strain in echocardiography: a test-retest study. J Am Soc Echocardiogr 2023;36:788–99. 36933849 10.1016/j.echo.2023.02.017

[30] Olaisen S, Smistad E, Espeland T, Hu J, Pasdeloup D, Østvik A et al Automatic measurements of left ventricular volumes and ejection fraction by artificial intelligence: clinical validation in real-time and large databases. Eur Heart J—Cardiovasc Imaging 2024;25:383–95. 37883712 10.1093/ehjci/jead280 PMC11024810

[31] Ouyang D, He B, Ghorbani A, Yuan N, Ebinger J, Langlotz CP et al Video-based AI for beat-to-beat assessment of cardiac function. Nature 2020;580:252–6. 32269341 10.1038/s41586-020-2145-8 PMC8979576

[32] Song J, Yao Y, Lin S, He Y, Zhu D, Zhong M. Feasibility and discriminatory value of tissue motion annular displacement in sepsis-induced cardiomyopathy: a single-center retrospective observational study. Crit Care 2022;26:220. 35851427 10.1186/s13054-022-04095-w PMC9295263

[33] Wang YH, Sun L, Li SW, Wang C-F, Pan X-F, Liu Y et al Normal reference values for mitral annular plane systolic excursion by motion-mode and speckle tracking echocardiography: a prospective, multicentre, population-based study. Eur Heart J—Cardiovasc Imaging 2023;24:1384–93. 37530466 10.1093/ehjci/jead187 PMC10531139

[34] Støylen A, Mølmen HE, Dalen H. Regional motion of the AV-plane is related to the cardiac anatomy and deformation of the AV-plane. Data from the HUNT study. Clin Physiol Funct Imaging 2023;43:453–62. 37395325 10.1111/cpf.12845

[35] Freitas-Ferraz AB, Bernier M, Vaillancourt R, Ugalde PA, Nicodème F, Paradis J-M et al Safety of transesophageal echocardiography to guide structural cardiac interventions. J Am Coll Cardiol 2020;75:3164–73. 32586591 10.1016/j.jacc.2020.04.069

[36] Ramalingam G, Choi SW, Agarwal S, Kunst G, Gill R, Fletcher SN et al Complications related to peri-operative transoesophageal echocardiography—a one-year prospective national audit by the association of cardiothoracic anaesthesia and critical care. Anaesthesia 2020;75:21–6. 31236918 10.1111/anae.14734

