# Supplementary material for: Continuous three-dimensional transesophageal echocardiography and deep learning for perioperative monitoring of left ventricular longitudinal function
Source: Eur Heart J Imaging Methods Pract. 2025 May 2;3(1):qyaf052. doi: 10.1093/ehjimp/qyaf052 (PMC12092336; doi:10.1093/ehjimp/qyaf052)
Supplement: qyaf052_Supplementary_Data [file qyaf052_supplementary_data.zip › Video 1 caption EHJ IMP.docx]

**Video 1 –** An example case demonstrating a bias (-1.6 mm) between 3D autoMAPSE (5.5 mm) and 3D manual MAPSE (7.1 mm) that was similar to the overall bias observed in this study (-1.4 mm). The bias may not be explained by poor landmark detection because the image quality and mitral annular segmentation were both excellent. Abbreviations: 3D autoMAPSE, automatic measurements of mitral annular plane systolic excursion by 3D transesophageal echocardiography; MAPSE, mitral annular plane systolic excursion.
