# Supplementary figures and images for: Continuous three-dimensional transesophageal echocardiography and deep learning for perioperative monitoring of left ventricular longitudinal function
Source: Eur Heart J Imaging Methods Pract. 2025 May 2;3(1):qyaf052. doi: 10.1093/ehjimp/qyaf052 (PMC12092336; doi:10.1093/ehjimp/qyaf052)

Bias -1.6 mm despite excellent  
detection and image quality

3D AutoMAPSE: 5.5 mm

3D Manual MAPSE: 7.1 mm

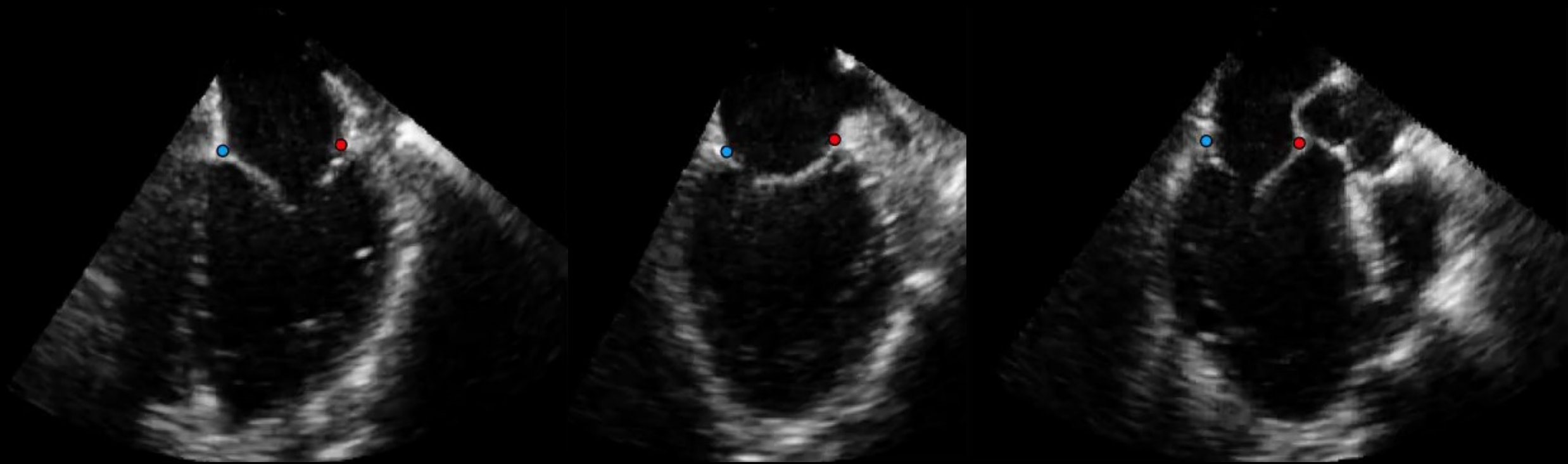

Supplement: qyaf052_Supplementary_Data [file qyaf052_supplementary_data.zip › Video 1 still.pdf]
